# Socio-economic determinants and environmental hygiene factors of female caregiver burden in two selected low-income communities in Cape Town, South Africa

**DOI:** 10.11604/pamj.2019.34.80.16719

**Published:** 2019-10-11

**Authors:** Yakubu Almu-min Yakubu, De Wet Schutte

**Affiliations:** 1 Department of Environmental and Occupational Studies, Cape Peninsula University of Technology, Cape Town, South Africa

**Keywords:** Socioeconomic, physical health, caregiver burden, low-income, environmental hygiene

## Abstract

**Introduction:**

Studies on female caregiver burden and its determinants in relation to physical environment and social support in low-income and middle-income countries are limited. This study evaluates the relationship between caregiving physical environment, social support and well-being of female caregivers and care recipients in Cape Town, South Africa.

**Methods:**

One hundred (100) each of black African and coloured female caregivers from two different population settlements were randomly selected. Structured questionnaire was employed to gather information from caregivers. Description and correlation analyses were used to examine the association between health status of care recipients, environmental hygiene factors and female caregiver burden.

**Results:**

About 49.5% of the female caregivers were between 50-59 years and worked full-time (≥40 hours per week). Better hygiene environment and working conditions are major determinants of caregiver burden and care recipient's physical health. Better hygiene conditions in the kitchen and toilet significantly increased care recipients' and caregivers’ physical health (P<0.05). Diarrhoea was found to be significantly associated with poorer environmental hygiene. Over 50% of the reported diarrhoea cases were among care recipients with poorer kitchen and toilet hygiene. Bad environmental hygiene increased the risk of diarrhoea among care recipients and caregivers. Physical health of the care recipients and social grants influenced the burden on the female caregiver.

**Conclusion:**

Increase social grants and attention to environmental conditions of caregiving will improve the physical health and living standard of the care recipients and caregivers.

## Introduction

In South Africa, both empirical and anecdotal evidence posit that poverty and inequality continue to detract from what some sections of the population have referred to as a ‘miracle transition’ [1]. A visibly highly skewed and endemic pattern of distribution remains a socio-economic reality, reinforcing poverty and inequality. Undoing them in the periods of poor economic performance remains the major preoccupation of policymakers in the current democratic South Africa. The most recent planning documents, the National Development Plan (NDP) and Vision for 2030, are anchored by two fundamental objectives; the elimination of poverty and reduction of inequality, and the recognition that the country faces a triple challenge of poverty, inequality and unemployment. In response to these constraints, a plethora of strategies for poverty reduction and improved health of the population have been introduced since 1994. This includes, but not limited to: national economic and development policy frameworks, the general move towards comprehensive social protection and developmental welfare and the free primary health care. Despite its upper middle-income status, the country still faces persistent socio-economic difficulties that the South African government describes as its triple challenge (poverty, unemployment and inequality) with embedded health issues [2, 3].

The Alma Atta declaration in Moscow in September 1978 gave impetus to universal primary health care access globally. The declaration reaffirms amongst others that, health is a fundamental human right and emphasized the attainment of the highest level of health as the most important world-wide social goal. The realization of it requires the action of many other social and economic sectors to supplement the health sector [4]. With an aging population and public policies that limit accessible and affordable formal care services, informal caregivers, largely women (i.e. female caregivers), will continue bearing the overwhelming responsibility for home-based care in the community (i.e. family caregiving) and long-term care services provision but, significant resources in the country are still tied up in the private sector and not available for the majority of the population (non-white) who are largely poor, leading to major inequities in the health care facilities used by different population groups. This makes the progressive and complete realisation of the Alma Atta declaration still a dream in the country after many decades. In tackling these imbalances, the South African constitution mandates the state to make efforts towards the progressive realisation of the right to health for all. In the caregiving arena several definitions are suggested for a family on informal caregiver but, typically the majority of informal caregivers are women and are primarily members of the same family to whom care is given [5-7]. On the contrary, in a recent study done in Pakistan (Irfan, Irfan & Ansari 2017), though an isolated case, reported that the majority were males (54%) and females (46%). In tandem with the majority of the literature, caregivers are typically the people who provide unpaid care services for the aged or for people needing assistance with tasks in the home that may be physically, emotionally, socially or financially challenging and involve much time and energy for long periods of time [8, 9]. The rendering of these services mostly come with some inherent effects that could either be perceived as negative or positive. It has been documented by other studies that when it presents negative outcomes, it is termed as caregiver burden that results from the responsibilities, demands, difficulties, and negative psychological consequences of caring for relatives particularly for those with special needs [10]. Currently, there has been an upsurge in interest and attention in caregiver research with a postulation that this increase is the result of various factors, including a drive towards deinstitutionalisation aimed at seeing the mentally ill and disabled integrated back into the community. However, it has been long posited that improvements in medical technology also aid in decreasing morbid conditions and mortality, enabling people with congenital and chronic illnesses to live longer [11].

Worldwide informal caregiving serves as a critical extension of the formal health care system. Thus, family caregivers assist care recipients with their needs for daily life activities including getting food, bathing, laundry, toileting and sometimes administering medication [12, 13]. In both low and middle-income countries informal caregivers provide about half of the care needs of care recipients [14]. They are the caregivers who provide unremunerated care to the majority of the care recipient’s physical, emotional, financial, and social care needs at the household level in the community [15]. South Africa is no exception, where the majority of the care recipients still live in the communities depending on the family for their needs for activities of daily living. In the African context, culturally the younger ones are to provide care to the aged and the care they received is returned to them when they grow old. As a result of the slowly demising of the collectivism due to increasing high costs of living coupled with unfavourable global economic conditions and many other factors, there is increasing demise of collectivism hence increasing stress levels giving rise to caregiving burden. Stress is not seen as inherent in the event itself, but rather conceptualized as a function of the response of the distressed and refers to the residue of tensions generated by the stressor which remain unmanaged [16]. Other researchers [8] argue that the chronic and demanding nature of family caregivers in the community, especially in poverty stricken households, can lead to a high degree of stress for caregivers (caregiver burden) and pressure on household and environmental health resources [17]. The problem is compounded with advanced age that comes with associated health concerns and needs for environmentally hygienic living conditions. The well-being of dependent members in a household is very crucial and the role played by informal caregivers cannot be underestimated. However, studies on female caregiver burden and its determinant in relation to physical environment and health in low-income and middle-income countries are limited. The understanding of caregiver burden and its factors and difficulties in low or middle-income countries, would help in developing appropriate strategies and interventions targeting improving the well-being of the community caregivers. In this case, the study seeks to bridge the gap by evaluating the relationship associated with the physical environment of caregiving household and the well-being of caregivers and care recipients within two selected community in Cape Town, South Africa.

## Methods

**Study design, setting and data source:** this study adopted the Stress Process Model (SPM) [18] that is largely consistent with other models such as the Lazarus and Folkman's model (1984) that provides a framework for explaining the processes involved when a person attempts to cope with stressful events. These models postulate that when individuals are confronted with a stressor, they evaluate the potential threats by making a primary appraisal that then integrates their judgement regarding the significance of the event (e.g. stressful or not stressful, negative or positive, controllable or uncontrollable). Thereafter, individuals make a secondary appraisal. The existing stress and coping models in the caregiving research tend to concentrate on six components including: context/demographic variables (e.g. gender, race, age, and relationship to recipient), demands on caregiver (e.g. recipient's functional abilities and time spent caring), appraised stressors associated with the caregiving situation (e.g. financial strain), personal demands (e.g. work status, family conflict, privacy), caregiver appraised buffers (e.g. active coping, social support), and long-term consequences (e.g. emotional distress, physical health outcomes). However, the majority of the literature focuses only on a subset of these categories of constructs with little account of the factors in the caregiving environment. Consequently, the measurement of these constructs is often limited to only a few of these variables.

This study takes an in-depth look at the relationships between caregiving burden and selected exogenous variables such as caregiver socio-economic status (SES), environmental health factors and the caregiver recipient health status. The study was carried out in two low-income different socio-cultural communities in Cape Town: New Rest in Gugulethu, and New Woodlands in Mitchells Plain. These communities were predominantly black or African dominant and coloured dominant settlements living in government subsidised housing. From each community, 100 female caregivers were selected through a systematic random sampling (SRS) procedure, giving a total of 200 female caregivers in this study. Data were collected according to the constructs of the SPM [18] with the focus on caregiver burden and health conditions of both caregiver and care recipients. A data collection instrument (questionnaire) was designed and used to collect the information through structured interviews with the main/primary female caregiver in each household. A pilot survey preceded the main study to test the study instrument for face validity and reliability in the questions.

**Inclusion criteria:** the respondents were the main/primary female caregivers who were present, willing and able to give informed consent. A caregiver was defined as having an elderly person and/or a non-biological childcare recipient under her care and living in a formal (government subsidised housing) settlement. Any sampled dwelling units that blend into shanty or shack areas were excluded. Also, all such dwelling units that formed part of the pilot study were excluded in the main study. A standard replacement procedure was employed in cases where a selected household did not qualify to be included in the study.

**Variables and measurement:** the outcome variables were physical health of care recipients (FHCR) and female caregiver burden (FCBur). The predictor variables are environmental hygiene, age, income, education, chronic diseases, government social grant and many others.

**Data analysis:** the study was carried out in 2014 with both objective and subjective measures through the use of a fully structured questionnaire in a face-to-face interview setting. Female caregiver burden was measured using self-report information from the participating caregivers. For this purpose, eight questions were used to assess financial strain, lack of privacy, sleep disturbance, physical strain, change in lifestyle, insufficient level of funds, suffered social life and no control over one's life. These elements were all measured on a 5-point Likert scale (1= strongly agree to 5= strongly disagree). After conducting reliability analysis (Cronbach's alpha= 0.XX), these items were transformed into a composite score (caregiver strain/burden) by determining the mean for all the items. The lowest caregiver strain was scored as X and the highest caregiver strain was scored as Y, with the mean caregiver strain XX (SD=YY). Functional status of the caregiver was assessed by using the activities of daily living and instrumental activities of daily living. The activities of daily living included difficulties caregivers experienced with feeding, cooking, dressing, bathing and washing the clothes of care recipients.

The instrumental activities of daily living included user needs of care recipients (i.e. wheel chair, spectacles, walking stick and transport). The activities of daily living and instrumental activities of daily living scores were created by adding the items in each of the functional status assessments. A higher score indicated a more dependent functional status. Further, a principal component factor analysis was performed, and it showed that each of the items for activities of daily living and instrumental activities of daily living measured one latent variable. Descriptive statistics were used to show the socio-economic characteristics of caregivers. Chi-square tests were used to show the association between socio-economic or background variables, the environmental hygiene factors of the caregiving environment (i.e. kitchen hygiene and toilet hygiene) and care recipients diarrhoea cases and physical health. Correlation analysis was used to examine the correlation between a set of variables and female caregiver burden. The data were analysed using SPSS version 22.

**Ethics approval and consent to participate:** the ethics committee of the Faculty of Applied Sciences of Cape Peninsula University of Technology (CPUT) provided the ethical clearance for this study (Ref 07/2013). Furthermore, each individual female caregiver in the study completed a consent form.

## Results

**Description analysis:** the reliability test showed that the Cronbach alpha for items of activities of daily living was 0.909 and that of instrumental activities of daily living was 0.836. There were 200 female caregivers with an average age of 47.9 years (standard deviation (SD)= 11.7 years). A greater proportion of the caregivers were between 50 years and 59 years old. All caregivers had some form of formal education with the least having completed Grade 1 education. Majority of the caregivers had some secondary education (Grade 8-11). About 65% of the caregivers were either married or had ever married. More than 50% of the caregivers earned less than R1001 (Table 1). The majority of the caregivers (72.1%) were reported to receive social grant from the government.

**Table 1:** Descriptive analysis of socio-economic characteristics of caregivers

Characteristics and profile		
Mean age in years (SD)	47.9 (12)	
Population	% (100)	N=200
**Education**		
≤Grade 7/Standard 5	10.5	21
Grade 8 − 11	54.0	108
Standard 10/(Grade 12)	31.0	62
Higher	4.5	
**Age Group (years)**		9
<30	11.5	23
30 − 49	23.0	66
50 – 59	49.5	99
60 and above	6.0	
**Income**		12
R0 – R1000	58.1	116
R1001 – R2000	39.3	79
R2001 and above	2.6	5

**Environmental hygiene and physical health analysis:** a statistically significant relationship was found between the environmental health status of the home and the physical health of care recipients (Table 2) (P<0.05). Better kitchen and toilet hygiene are significantly associated with improved physical health. About 84% of the caregivers reported better physical health of care recipient compared to 16% being worse. Similarly, 78% and 76.5% of the caregivers operate under good kitchen and toilet hygiene (Table 2). Environmental hygiene (kitchen or toilet hygiene) is significantly associated with the number of diarrhoea cases reported in care recipients (Table 3). The better kitchen and toilet hygiene, the lower the number of reported cases of diarrhoea among care recipients. More than 50% of caregivers under bad environmental hygienic condition reported diarrhoea cases among the care recipients within 4 weeks preceding this study (Table 3). However, 31% of diarrhoea cases were reported during the study period. A further 56.5% of the care recipients reported having at least a chronic disease.

**Table 2:** Environmental hygiene and physical health of care recipients

Environmental health	Physical Health				*X^2^*	P-Values
	Very Good	Good	Bad	Total		
	n=64	n=104	n=32			
**Kitchen Hygiene**						
Bad	2.0	18.0	2.0	**22**	20.580	<0.001*
Good	30.0	34.0	14.0	**78**		
Toilet Hygiene						
Bad	3.0	18.5	2.0	**23.5**	17.694	<0.001*
Good	29.0	33.5	14.0	**76.5**		

* Significant provability value (p-Value)

**Table 3:** Reported environmental hygiene and diarrhoea cases of care recipients reported

Environmental health	Diarrhoea Cases			P-Values
	Yes	No	Total	
**Kitchen Hygiene (%)**				
Bad	54.0	24.0	78	<0.001*
Good	15.0	7.0	22	
**Toilet Hygiene (%)**				
Bad	53.5	23.0	76.5	<0.001*
Good	15.5	8.0	13.5	
**Disease (%)**				
Diarrhoea	31	69	100	<0.001*
Chronic	56.5	43.5	100	0.049*

* Significant provability value (p-Value)

**Correlation analysis:** the findings suggest that there were significant positive associations between FCBur and age, income status, chronic diseases, diarrhoea, social grants, kitchen hygiene, toilet hygiene and physical health of care recipients (p<0.05) (Table 4). However, education was not a significant determinant of caregiver burden (r= 0.014, p>0.05). Toilet hygiene interacted with diarrhoea to increase the caregiver burden. Chronic diseases combined with toilet hygiene, care recipient health and social grant increased caregiver burden (r= 0.033, p<0.05) (Table 4).

**Table 4:** Pearson’s correlations for the determinants of caregiver burden

		Caregiver burden	1	2	3	4	5	6	7	8	9	10	11
Determinant													
**1**	**Age**												
1	Age	0.179*	-										
2	Level of education	0.014	-0.259**	-									
3	Income status	0.149*	0.314**	-0.107	-								
4	Population group	-0.294**	-0.497**	0.243**	-0.724**	-							
5	Number of hours of care	-0.248**	-0.410**	0.154*	-.730**	0.912**	-						
6	Chronic diseases	0.172*	0.323**	-0.150*	-0.056	0.061	0.099	-					
7	Diarrhoea	0.111*	-0.126	0.158*	-0.143*	0.202**	0.147*	-0.097	-				
8	Social grants	0.453**	0.441**	-0.185**	0.296**	-0.438**	-0.342**	0.164*	-0.105	-			
9	Kitchen hygiene	0.206**	0.234**	-0.120	0.236**	-0.410**	-0.372**	-0.037	-0.015	0.252**	-		
10	Toilet Hygiene	0.244**	0.314*	-0.102	0.006*	0.422	-0.061	0.033*	341**	0.012*	0.121	-	
11	Care recipients’ physical health status	0.459**	0.220**	-0.011	0.222**	-0.267**	-0.223**	0.149*	-0.075	0.491**	0.022	0.321**	-

## Discussion

The SPM was used to evaluate caregiving environment hygiene, physical health and determinants of FCBur within two low-income communities living in government housing in Cape Town, South Africa. The findings of the study indicated that the major factors impacting on the care recipient’s physical health and caregiver burden were environmental hygiene, social grants, and chronic diseases. Age and income were also determinants of caregiver burden. Education was found not to play a significant impact on caregiver burden, however, caregiver with higher education and receiving social grants from government, lessen caregiver burden even though care recipient may have chronic diseases. Studies have demonstrated that caregivers, particularly the younger caregivers, often neglect their education, putting education on hold or even drop out entirely, which can impact their future career [2].

Many socio-economic and demographic determinants interact to increase the caregiver burden and care recipient health in-low income settings in Cape Town, South Africa. Our findings indicate that majority of the caregivers earned low-income (i.e. less than R1001 pm). This may be due to their low educational background, which cannot earn them a high paid employment. The low-income earned by the greater number of caregivers is a contributing factor of the significant burden on them. The findings support the study that the majority of the caregivers who suffered from financial difficulties while providing care also find it difficult in fulfilling other responsibilities such as marriage and employment [19, 20]. In support of this, empirical evidence [9, 12] buttressing the importance of resources indicated that when the stress resulting from care surpasses the resources available to cope with the caregiving demands caregiving burden occurs. Other studies suggest that informal caregivers have even worst experiences ranging from career retrogression, job loss, and marital disruption and to some extent crisis within family systems while they strive to create balance between caregiving and other responsibilities including those of gender [20].

Our finding shows that environmental health hygiene and physical health of care recipients which are significant determinants of caregiver burden is not consistent with previous finding that caregiving burden does not influence caregivers physical activities [10, 21]. The findings support anecdotal evidence suggesting that educated people and good income earners live in hygienically cleaner, more protected environments and are healthier compared with less affluent families [22]. The impact of the environmental hygiene, i.e. kitchen and toilet hygiene on both caregiver and care recipient collaborates earlier studies that greater satisfaction with life is largely related with important measures of life quality as reflected in the level of cleanliness and protective nature of the living environment [22]. The outcome that better environmental hygiene was associated with reduced reported cases of diarrhoea among care recipients and caregivers support previous study that domestic environment such as kitchen setting is a source of microbial cross contamination [23]. However, it presents health concerns in the demographic and socio-environmental living arrangements. It has been documented that in developing countries diarrhoeal diseases are a critical health condition due to the rapid increasing urbanization and the related environmental sanitation issues.

Empirical studies also buttressed that poor sanitation at the household or community levels contribute to approximately 94% of the four billion cases of diarrhoea occurring globally per annum [24-26]. Furthermore, it was reported that diarrhoeal disease is responsible for 4% (2 million deaths) of global mortality and causing about 1.3 million deaths in children annually [27]. Poverty is a known factor of the disease and mostly affects poverty-stricken populations characterised with low-income and low socio-economic status [28]. The caregivers in these communities should be given more education on environmental sanitation to be able to manage their hygiene conditions for better care recipient and caregiver health outcomes. The Governments of low and middle income countries need to speed up efforts to improve their health care system to enable them respond to caregiver demands and provide adequate resources for the family caregivers to cope with their care roles. After all, informal caregiving forms part of the health care sector of many countries for the purpose to improve the lives of all the citizenry. Given the fact that social grant is one of the determinants of caregiver burden, informal caregivers must formally be registered and grant given to them be increased to motivate them to give their best of care to the care recipients. Increased grant will also improve their standard of living of the caregiver as well as the care recipients.

**Strength and limitations:** the study on the services of a second major caregiver could help ameliorate the negative effect of caregiving on the main caregiver. This study also reinforces that the factors of physical environments are critical determining factors for healthy living for both caregivers and care recipients of the low-income settlements. Thus, a large study is needed involving many of such communities in Cape Town for policy interventions. The findings of this study cannot be generalised across all income bracket since the data captured was provided at a specific point in time as the caregiver’s current experience.

## Conclusion

Better hygiene environment and working conditions proved to be major determinants of caregiver burden and care recipient’s physical health. Poorer environmental household conditions increase the risk of the care recipients and even caregivers contracting diarrhoea. We therefore recommend that attention be paid to the environmental conditions under which the caregivers work in order to improve the physical health of the care recipients and caregiver. We also recommend that caregiving burden can be reduced by improving caregiver’s physical activities and should be considered as a separate core issue in planning interventions in the public care system in the country.

### What is known about this topic

- Caregiver burden has clinically been proved to have negative impact on the physical and mental health of the caregiver;
- Socio-demographical attributes of both the caregiver and care-recipient, and the perceived stress are causative factors of caregiver burden role;
- A female in the household often assumes the major caregiver role.

### What this study adds

- The physical health status of the care recipients is a significant contributor to caregiver burden;
- Poorer environmental condition increases the risk of getting diarrhoea in both caregiver and care recipient;
- Chronic diseases and insufficient social grants increase the burden of the caregiver and worsen the pride of care recipient.

## Competing interests

The authors declare no competing interests.
